# A machine learning-based predictive model for 48-week hepatitis B surface antigen seroclearance in chronic hepatitis B patients treated with pegylated interferon α-2b: prediction at week 24

**DOI:** 10.3389/fcell.2025.1734654

**Published:** 2025-11-26

**Authors:** Nan Kong, Kaixia Wang, Yiling Wang, Shike Lou, Luocheng Zhou, Tao Wang, Zhili Tan, Lihong Qu

**Affiliations:** Department of Infectious Diseases, Shanghai East Hospital, Tongji University School of Medicine, Shanghai, China

**Keywords:** chronic hepatitis B, HBsAg seroclearance, predictive model, machine learning, clinical utility

## Abstract

**Background:**

Chronic hepatitis B (CHB) is an infectious disease mainly affecting the liver, caused by the hepatitis B virus (HBV). In the treatment of CHB, pegylated interferon α-2b (PEG-IFNα-2b) is one of the important therapeutic options. However, there are significant individual differences in patients’ responses to this treatment and only a few patients can achieve hepatitis B surface antigen (HBsAg) seroclearance. Therefore, an effective method to identify patients with a high likelihood of favorable response at an early stage is urgently needed.

**Methods:**

In this study, we analyzed data from CHB patients who received antiviral treatment with PEG-IFNα-2b and completed 48 weeks of follow-up in the “OASIS” Project. Patients were divided into the seroclearance group and the non-seroclearance group based on whether HBsAg seroclearance was achieved at week 48.Five distinct machine learning feature selection algorithms were used to identify the optimal predictive variables for HBsAg seroclearance. These key variables were then incorporated into 12 machine learning algorithms to build predictive models for HBsAg seroclearance. The best-performing model was selected, and its performance was evaluated.

**Results:**

A total of 680 subjects were included in this study, comprising 165 in the 48-week seroclearance group and 515 in the 48-week non-seroclearance group. Through five different machine learning feature selection algorithms, 11 variables were identified and used to construct 12 distinct machine learning models. Comparative analysis of these models, based on the Area Under the Receiver Operating Characteristic Curve (AUC) and Decision Curve Analysis (DCA) results from the training set, indicated that the Random Forest model was the optimal model for predicting HBsAg seroclearance.

**Conclusion:**

The Random Forest model effectively predicted the 48-week HBsAg seroclearance rate using indicators measured at 24 weeks of PEG-IFNα-2b therapy. This model can provide a reliable reference for optimizing clinical treatment strategies.

## 1 Introduction

CHB is an infectious liver disease caused by persistent hepatitis B virus (HBV) infection for more than 6 months. According to the WHO’s 2024 Global Hepatitis Report, it affected 254 million people globally in 2022, with approximately 1.2 million new infections and 1.1 million deaths annually, primarily due to cirrhosis and hepatocellular carcinoma (Burki, 2024).In China, the CHB epidemic remains a serious concern. A nationwide study revealed approximately 75 million individuals living with chronic HBV infection. Despite this progress from 120 million in 1992, the prevention and control situation remains challenging. Among these 75 million, an estimated 30 million are unaware of their status, and 17 million require antiviral treatment, yet only three million are receiving it (Hui et al., 2024).These critical gaps in diagnosis and treatment expose a vast number of individuals to a high risk of disease progression to severe complications, including cirrhosis and hepatocellular carcinoma (HCC), which severely diminish quality of life and create a heavy socioeconomic burden (Jian et al., 2025).

The achievement of HBsAg seroclearance, regarded as a “functional cure” for CHB, significantly reduces the risk of cirrhosis and HCC, thereby establishing it as a primary therapeutic goal. As a key drug for immunomodulatory treatment of CHB, PEG-IFNα-2b is widely used in eligible patients due to its advantages of limited treatment duration and potential curability. However, treatment response exhibits substantial inter-individual variation, and the rate of HBsAg seroclearance remains low. Moreover, the current clinical paradigm requires awaiting 48-week outcomes to assess final response, which can result in ineffective treatment for non-responders, escalating both healthcare costs and the risk of adverse events (Hong et al., 2022). Early identification of patients with a high likelihood of HBsAg seroclearance during PEG-IFNα-2b treatment is crucial for optimizing individualized CHB treatment. Currently, most domestic and international studies continue to focus on baseline or early-treatment (e.g., week 12) predictors (Lee et al., 2025; Ren et al., 2021; Rijckborst et al., 2010; Sonneveld et al., 2010; Ye et al., 2024). However, week 24 of therapy is a well-established key timepoint for assessing response to interferon-based regimens and determining whether to adjust treatment strategies (Hong et al., 2022). Developing a robust predictive model at this juncture, integrating multidimensional data such as dynamic liver function indices and serological virological markers, can provide clinicians with a practical tool at this critical decision-making window. This enables early warning for potential non-responders, allowing timely strategy modifications, while reinforcing treatment confidence for potential responders, ultimately advancing personalized and precision management of CHB.

In the era of big data and artificial intelligence, machine learning (ML)—a concept pioneered by Arthur Samuel—enables computers to simulate human learning and predict outcomes through mathematical modeling (Deo, 2015).ML encompasses a wide range of mathematical models, such as Decision Trees, Random Forests, Support Vector Machines (SVM), k-Nearest Neighbors (kNN), Neural Networks, and Naive Bayes (NB) (Asnicar et al., 2024; Kotsiantis et al., 2006; Sim et al., 2023).ML techniques are particularly adept at processing high-dimensional clinical data and uncovering complex relationships to build reliable ML models. (Fan et al., 2023; Lee et al., 2023; Xue et al., 2025).

Based on the multi-center, prospective, real-world OASIS project, this study collected clinical data from CHB patients treated with PEG-IFNα-2b. The aim was to develop and validate a ML-based prediction model that utilizes accessible indicators at 24 weeks of treatment—such as quantitative HBsAg and virological markers (e.g., HBV DNA load)—to accurately predict the HBsAg seroclearance rate at 48 weeks. The resulting model is expected to provide clinicians with a decision-support tool for the early identification of patients who are most likely to respond favorably to PEG-IFNα-2b therapy, thereby reducing ineffective treatments and advancing the optimization of individualized CHB management.

## 2 Materials and methods

### 2.1 Participants

This study utilized data from the “China Hepatitis Prevention and Treatment Foundation - Project to Reduce Hepatocellular Carcinoma Incidence in Chronic Hepatitis B Patients” (the OASIS project, NCT04896255). This large, multicenter, prospective, real-world study enrolled CHB patients from 32 provinces across China, including treatment-naïve individuals, those previously treated with PEG-IFNα-2b, and those with nucleos(t)ide analogues (NAs) experience. Participants received either a PEG-IFNα-2b-based regimen (as monotherapy or in combination with NAs) or NAs monotherapy and are being followed for a planned 5-year period. This study was approved by the Ethics Committee of Shanghai East Hospital, Tongji University (Approval Number: [2020] Pre-review No. (157)). Written informed consent was obtained from all participants. The data were sourced from the OASIS project, with data cleaning and quality control handled by National Medical Center of Infectious Diseases (NMCID) Liver Diseases Research Group.

The specific inclusion criteria were as follows: (1) Chronic HBV infection, defined as: positivity for HBsAg for more than 6 months, or positivity for HBsAg for less than 6 months but with a liver biopsy within the past year meeting the pathological criteria for chronic hepatitis B, with other liver diseases excluded. (2) Age between 18 and 80 years, inclusive, irrespective of gender. (3)Treatment with PEG-IFNα-2b-based antiviral therapy. (4)Completion of the 48-week follow-up.

**FIGURE 1:**
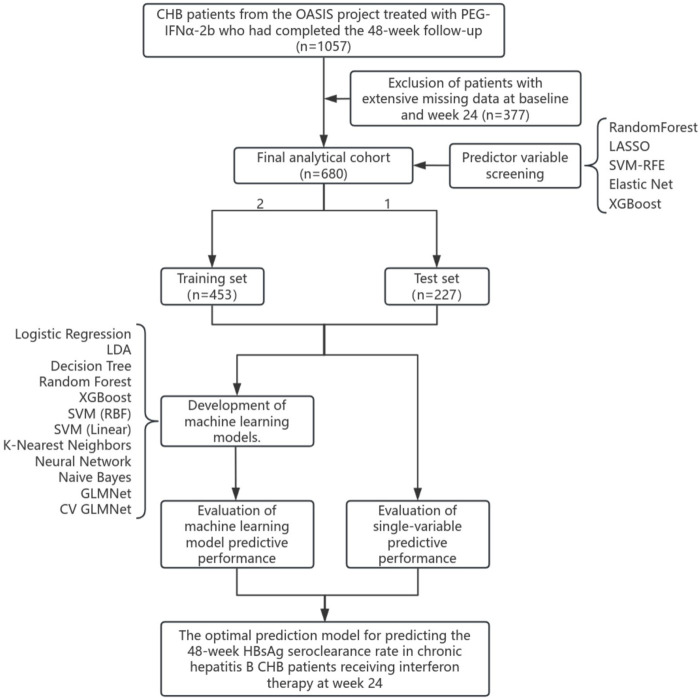
Study flowchart.

Of the 1,057 eligible CHB patients, 377 were excluded from the final analysis due to extensive missing data at baseline or the 24-week time point. Consequently, the final analytical cohort consisted of 680 patients.A flowchart detailing the study design and patient selection process is presented in Figure 1.

### 2.2 Data preprocessing and feature engineering

The clinical dataset for this study comprised demographic and clinical characteristics—including age, gender, height, weight, body mass index (BMI), cirrhosis status, and family history—of CHB patients treated with PEG-IFNα-2b. It also included serial serological parameters measured at both baseline and 24 weeks (6 months) of treatment. These parameters covered virological markers (HBV DNA, HBsAg, HBeAg, HBeAb, HBcAb), liver function indices (ALT, AST, TBIL, DBIL, ALP, GGT, ALB, GLO), and routine blood test results (PLT, NEU, Hb).The initial data processing steps included: handling missing values, applying logarithmic transformation to viral load markers (HBV DNA, HBsAg, HBeAg, HBeAb, HBcAb), and deriving kinetic variables (HBsAg_desc = baseline HBsAg level - week 24 HBsAg level; HBeAg_desc = baseline HBeAg level - week 24 HBeAg level; HBeAb_desc = baseline HBeAb level - week 24 HBeAb level; HBcAb_desc = baseline HBcAb level - week 24 HBcAb level). Categorical variables (gender, cirrhosis, family history) were factor-encoded. After excluding patients with excessive missing data, the final dataset contained 31 predictor variables. The quantitative results for HBV DNA and HBsAg were log_10_-transformed during data preprocessing.

### 2.3 Feature selection using machine learning

To identify robust predictors of treatment response, we employed five distinct feature selection algorithms. (1)Random Forest (RF): we calculated mean decrease in Gini impurity using the randomForest package to rank feature importance (Chen et al., 2024; Hosseini Sarkhosh et al., 2022). (2)Least Absolute Shrinkage and Selection Operator (LASSO): using 5-fold cross-validation via glmnet, we selected non-zero coefficients at λmin to retain features with strongest associations (Friedman et al., 2010; Simon et al., 2011). (3)Support Vector Machine-Recursive Feature Elimination (SVM-RFE): we implemented backward feature elimination with linear SVM kernel and 5-fold cross-validation (Huang et al., 2018). (4)Elastic Net: combining L1 and L2 regularization (α = 0.5), we identified features with persistent coefficients across cross-validation folds (Hughey and Butte, 2015). (5) XGBoost: we utilized gradient boosting with xgboost package, ranking features by gain importance metric which quantifies the total reduction of loss contributed by each feature. The model was configured with multi:softmax objective, maximum depth of 3, learning rate of 0.1, and 100 boosting rounds with early stopping (Chen and Guestrin, 2016; Liang et al., 2025).

Features consistently ranked highly across multiple methods were prioritized for model construction.

### 2.4 Predictive model development

We trained 12 diverse classifiers using the mlr3 framework, including (1) Linear models: Logistic Regression, Linear Discriminant Analysis; (2) Tree-based models: Decision Tree, Random Forest, XGBoost; (3) Kernel methods: SVM (linear/RBF kernels); (4) Distance-based: k-Nearest Neighbors; (5)Neural networks: Single-hidden-layer Network; (6) Probabilistic: Naïve Bayes; (7) Regularized regression: GLMNet,CV GLMNet. All models were configured for probability prediction and trained on a stratified 2:1 train-test split. Hyperparameters used default values from respective mlr3learners implementations. Model performance was assessed using: Discrimination metrics: Area under ROC curve (AUC), accuracy, sensitivity, specificity, precision; F1-score; Calibration: Precision-Recall curves and net benefit analysis via Decision Curve Analysis (DCA); Stability: Comparative training-test performance and ranking consistency across metrics.

We implemented k-fold cross-validation during feature selection phases and held-out test set validation for final model assessment. Additionally, for optimal models, we performed SHapley Additive exPlanations (SHAP) analysis using DALEXtra to compute Shapley values for feature contribution quantification. Besides, We also performed univariate ROC analysis of single features and compared with ML models. This comprehensive framework ensured robust feature selection, validated model performance, and facilitated clinical interpretation of predictive factors for interferon response.

### 2.5 Statistical analyses

Based on HBsAg seroclearance status at week 48, patients were categorized into “Seroclearance” and “Non-Seroclearance” groups. Continuous variables with a normal distribution are presented as mean (standard deviation, SD) and compared using the independent samples t-test. Non-normally distributed variables are expressed as median (interquartile range, IQR) and compared using the Mann-Whitney U test. Categorical variables are summarized as percentages and compared using the chi-square test. All statistical analyses were performed with R software (version 4.5.1).The significance level (α) was set at 0.05, and a result with *P* < 0.05 was considered statistically significant.

## 3 Results

### 3.1 Characteristics of the study

Of the 680 CHB patients included in this study, 165 (24.3%) were classified into the seroclearance group and 515 (75.7%) into the non-seroclearance group. The data preprocessing pipeline began with the enumeration of missing values across all observations. Subsequent analysis was confined to clinical data from baseline (0W) and the 6-month (24W) time points. After excluding patients with a substantial amount of missing data, the final dataset comprised the following 31 predictor variables: Age, Gender, BMI, Liver Cirrhosis (LC), Family history, HBsAg (0W), HBsAg (24W), HBsAg_desc, HBeAg(0W), HBeAg(24W), HBeAg_desc, HBeAb (0W), HBeAb (24W), HBeAb_desc, HBcAb (0W), HBcAb (24W), HBcAb_desc, ALT (0W), ALT (24W), AST (0W), AST (24W), GGT (0W), GGT (24W), TBIL (0W), TBIL (24W), DBIL (0W), DBIL (24W), ALP (0W), ALP (24W), and PLT (0W),PLT (24W) (Supplementary Table S1).

The baseline characteristics of two groups are presented in Table 1. Differences between groups were assessed using the Chi-square test and the Mann-Whitney U test, with a statistical significance level set at α = 0.05. Significant baseline differences (all P < 0.05) between groups were observed in: LC (4.2% vs. 9.7%,*P* = 0.041), Family history (22.4% vs. 31.8%,*P* = 0.027) HBV DNA (median 0.00 vs. 1.62 log_10_ IU/mL, *P* = 0.034), HBsAg (median 1.86 vs. 3.08 log_10_ IU/mL, *P* < 0.001), HBeAg(median 0.40 vs. 0.52 COI, P < 0.001), HBeAb (median 0.03 vs. 0.18 COI, *P* = 0.001), HBcAb (median 3.29 vs. 3.28 COI, *P* = 0.012), ALT (median 26.70 vs. 31.00 U/L, *P* = 0.004), AST (median 23.70 vs. 27.00 U/L, *P* = 0.006), GGT (median 19.00 vs. 24.00 U/L, *P* = 0.001), ALB (median 46.00 vs. 45.10 g/L, *P* < 0.001), and Hb(median 148.00 vs. 153.00 g/L, *P* = 0.005). The laboratory parameters of two groups after 24 weeks of PEG-IFNα-2b treatment are presented in Table 2. Statistical comparisons were performed using the Chi-square test and Mann-Whitney U test, with the significance level set at α = 0.05. The analysis revealed statistically significant differences (all *P* < 0.05) in the following variables at week 24:HBsAg (median 0.00 vs. 2.47 log_10_ IU/mL, *P* < 0.001), HBeAg(median 0.37 vs. 0.45 COI, *P* < 0.001), HBeAb (median 0.04 vs. 0.26 COI, *P* < 0.001), and Hb(median 131.00 vs. 135.00 g/L, *P* = 0.006).

**TABLE 1:** Baseline characteristics and intergroup comparisons of the 680 patients.

Items	Total patient (n = 680)	Seroclearance group (n = 165)	Non-seroclearance group (n = 515)	*p*
Age (years)	38.00 [32.00, 46.00]	38.00 [32.00, 45.00]	38.00 [32.00, 46.00]	0.681
Height (cm)	170.00 [163.00, 174.00]	170.00 [162.00, 174.00]	170.00 [163.50, 175.00]	0.282
Weight (kg)	69.00 [59.00, 75.00]	67.00 [56.00, 74.00]	70.00 [60.00, 75.00]	0.06
BMI(kg/m^2^)	23.53 [21.25, 25.12]	23.15 [20.96, 24.91]	23.66 [21.47, 25.26]	0.088
Gender (%)				
Male	467 (68.7)	104 (63.0)	363 (70.5)	0.089
Female	213 (31.3)	61 (37.0)	152 (29.5)	
LC (%)				
Yes	57 (8.4)	7 (4.2)	50 (9.7)	0.041
No	623 (91.6)	158 (95.8)	465 (90.3)	
Family history (%)				
Yes	201 (29.6)	37 (22.4)	164 (31.8)	0.027
No	479 (70.4)	128 (77.6)	351 (68.2)	
HBVDNA (log_10_IU/ml) (0W)	1.34 [0.00, 3.89]	0.00 [0.00, 3.08]	1.62 [0.00, 4.26]	0.034
HBsAg (log_10_IU/ml) (0W)	2.81 [1.90, 3.47]	1.86 [0.86, 2.58]	3.08 [2.30, 3.65]	<0.001
HBeAg (COI) (0W)	0.49 [0.10, 1.30]	0.40 [0.09, 0.56]	0.52 [0.14, 1.99]	<0.001
HBeAb (COI) (0W)	0.11 [0.01, 1.55]	0.03 [0.01, 1.20]	0.18 [0.01, 1.76]	0.001
HBcAb (COI) (0W)	3.28 [3.08, 3.44]	3.29 [3.18, 3.49]	3.28 [3.04, 3.42]	0.012
ALT (U/L) (0W)	30.00 [19.00, 50.40]	26.70 [15.70, 42.30]	31.00 [20.00, 51.90]	0.004
AST (U/L) (0W)	26.00 [21.00, 34.19]	23.70 [19.80, 30.60]	27.00 [21.00, 35.30]	0.006
Tbil (μmol/L) (0W)	14.18 [10.90, 18.90]	13.50 [10.80, 19.20]	14.20 [10.94, 18.70]	0.948
Dbil (μmol/L) (0W)	3.00 [2.20, 4.20]	3.10 [2.30, 4.22]	3.00 [2.20, 4.16]	0.472
ALP (μmol/L) (0W)	80.00 [65.15, 97.45]	77.00 [64.00, 90.70]	80.85 [66.85, 98.08]	0.111
GGT (U/L) (0W)	23.00 [15.00, 36.54]	19.00 [13.00, 31.00]	24.00 [16.00, 38.00]	0.001
ALB (g/L) (0W)	45.50 [43.48, 47.32]	46.00 [44.00, 48.00]	45.10 [43.20, 47.00]	<0.001
GLO (g/L) (0W)	29.00 [26.35, 31.00]	28.10 [26.00, 30.85]	29.00 [26.40, 31.35]	0.163
NEU(10^9^/L) (0W)	2.85 [2.16, 3.71]	2.66 [2.06, 3.39]	2.90 [2.20, 3.73]	0.084
PLT (10^9^/L) (0W)	204.00 [164.00, 245.00]	206.00 [161.00, 251.00]	203.00 [165.50, 243.50]	0.984
Hb(g/L) (0W)	152.00 [137.00, 162.00]	148.00 [133.00, 159.00]	153.00 [139.00, 162.50]	0.005

**TABLE 2:** Comparison between the two groups after 24 weeks of treatment in the 680 patients.

Items	Total patient (n = 680)	Seroclearance group (n = 165)	Non-seroclearance group (n = 515)	*p*
HBVDNA (log_10_IU/ml) (24W)	0.00 [0.00, 0.00]	0.00 [0.00, 0.00]	0.00 [0.00, 0.00]	N/A
HBsAg (log_10_IU/ml) (24W)	2.10 [0.43, 3.14]	0.00 [0.00, 0.40]	2.47 [1.57, 3.37]	<0.001
HBeAg(COI) (24W)	0.44 [0.09, 0.91]	0.37 [0.08, 0.49]	0.45 [0.09, 1.52]	<0.001
HBeAb (COI) (24W)	0.12 [0.01, 1.40]	0.04 [0.01, 0.93]	0.26 [0.02, 1.50]	<0.001
HBcAb (COI) (24W)	3.23 [3.03, 3.42]	3.23 [3.07, 3.44]	3.23 [3.01, 3.41]	0.328
ALT (U/L) (24W)	45.20 [30.00, 71.55]	48.80 [29.42, 80.20]	45.00 [30.20, 68.00]	0.416
AST (U/L) (24W)	44.00 [32.27, 65.00]	46.20 [33.70, 70.00]	43.00 [32.00, 63.00]	0.192
Tbil (μmol/L) (24W)	12.80 [10.30, 15.97]	12.50 [9.94, 15.45]	13.06 [10.41, 16.20]	0.107
Dbil (μmol/L) (24W)	3.20 [2.50, 4.22]	3.20 [2.53, 4.39]	3.20 [2.50, 4.20]	0.512
ALP (μmol/L) (24W)	83.00 [70.85, 98.83]	81.05 [70.92, 97.07]	84.00 [70.75, 99.00]	0.337
GGT (U/L) (24W)	50.10 [32.00, 91.00]	49.00 [32.00, 110.60]	51.00 [32.00, 87.17]	0.886
ALB (g/L) (24W)	44.00 [42.00, 45.41]	44.00 [42.00, 45.60]	44.00 [42.00, 45.39]	0.683
GLO (g/L) (24W)	29.00 [26.60, 32.00]	29.00 [26.40, 32.10]	29.00 [26.73, 32.00]	0.65
NEU(10^9^/L) (24W)	1.63 [1.25, 2.21]	1.61 [1.21, 2.03]	1.63 [1.27, 2.24]	0.215
PLT (10^9^/L) (24W)	116.00 [92.00, 152.00]	121.00 [96.00, 152.00]	114.00 [91.00, 152.00]	0.282
Hb(g/L) (24W)	134.00 [122.00, 146.00]	131.00 [119.00, 143.00]	135.00 [124.00, 147.00]	0.006

Using the mlr3 framework in R, we implemented a 2:1 random split of patients into training (n = 453) and validation (n = 227) sets, followed by 500 bootstrap iterations to enhance the robustness of model assessment.Comparative analysis of baseline and 24-week treatment characteristics between the training and validation sets (Tables 3, 4) showed that, aside from family history (35.2% vs. 26.7%, *P* = 0.027), ALT at week 24 (median 48.80 vs. 44.00 U/L, *P* = 0.023), AST at week 24 (median 46.20 vs. 42.20 U/L, *P* = 0.048), and GGT at week 24 (median 55.00 vs. 48.00 U/L, *P* = 0.013), no other indicators exhibited significant differences (*P* > 0.05). This indicates that the baseline characteristics of the two sets were overall well-balanced, supporting their use for model training and validation.

**TABLE 3:** Comparison of baseline characteristics between the training and test sets.

Items	Test set (n = 227)	Training set (n = 453)	*p*
Age	38.00 [32.00, 45.00]	38.00 [32.00, 46.00]	0.872
Height	170.00 [163.00, 175.00]	170.00 [163.00, 174.00]	0.764
Weight	69.00 [60.00, 75.00]	70.00 [58.00, 75.00]	0.894
BMI	23.39 [21.51, 25.08]	23.66 [21.03, 25.26]	0.967
Gender (%)			0.916
Male	157 (69.2)	310 (68.4)	
Female	70 (30.8)	143 (31.6)	
LC (%)			0.458
Yes	16 (7.0)	41 (9.1)	
No	211 (93.0)	412 (90.9)	
Family.history (%)			0.027
Yes	80 (35.2)	121 (26.7)	
No	147 (64.8)	332 (73.3)	
HBVDNA (log_10_IU/ml) (0W)	1.36 [0.00, 3.65]	1.32 [0.00, 4.08]	0.698
HBsAg (log_10_ IU/ml) (0W)	2.92 [2.03, 3.38]	2.76 [1.81, 3.54]	0.586
HBeAg(COI) (0W)	0.51 [0.10, 1.06]	0.48 [0.11, 1.40]	0.882
HBeAb (COI) (0W)	0.17 [0.02, 1.55]	0.08 [0.01, 1.53]	0.266
HBcAb (COI) (0W)	3.31 [3.15, 3.46]	3.26 [3.05, 3.42]	0.057
ALT (U/L) (0W)	29.90 [19.00, 47.76]	30.00 [19.00, 51.30]	0.86
AST (U/L) (0W)	26.00 [21.00, 33.98]	26.00 [21.00, 35.00]	0.7
Tbil1 (μmol/L) (0W)	14.50 [10.95, 19.57]	14.08 [10.90, 18.60]	0.266
Dbil1 (μmol/L) (0W)	3.17 [2.20, 4.27]	2.93 [2.20, 4.16]	0.226
ALP (μmol/L) (0W)	79.90 [66.07, 94.00]	80.00 [65.03, 98.07]	0.737
GGT (U/L) (0W)	24.00 [15.00, 36.70]	22.40 [15.00, 36.12]	0.589
ALB (g/L) (0W)	46.00 [43.50, 47.45]	45.39 [43.50, 47.00]	0.368
GLO (g/L) (0W)	28.70 [26.60, 31.15]	29.00 [26.17, 31.00]	0.835
NEU(10^9^/L) (0W)	2.90 [2.15, 3.71]	2.84 [2.18, 3.68]	0.812
PLT (10^9^/L) (0W)	201.00 [164.00, 240.50]	205.00 [164.00, 251.00]	0.51
Hb(g/L) (0W)	154.00 [137.50, 162.00]	151.00 [137.00, 161.00]	0.352

**TABLE 4:** Comparison of patient characteristics at 24 Weeks of treatment between the training and test sets.

Items	Test set (n = 227)	Training set (n = 453)	*p*
HBVDNA (log_10_IU/ml) (24W)	0.00 [0.00, 0.00]	0.00 [0.00, 0.00]	N/A
HBsAg (log_10_IU/ml) (24W)	2.18 [0.42, 3.07]	2.06 [0.44, 3.21]	0.818
HBeAg(COI) (24W)	0.41 [0.08, 0.84]	0.44 [0.09, 1.00]	0.291
HBeAb (COI) (24W)	0.21 [0.02, 1.45]	0.10 [0.01, 1.37]	0.248
HBcAb (COI) (24W)	3.26 [3.05, 3.44]	3.22 [3.01, 3.41]	0.192
ALT (U/L) (24W)	48.80 [32.80, 78.65]	44.00 [29.00, 66.90]	0.023
AST (U/L) (24W)	46.20 [34.00, 68.25]	42.20 [31.60, 63.00]	0.048
Tbil1 (μmol/L) (24W)	13.30 [10.65, 15.97]	12.71 [10.10, 15.97]	0.455
Dbil1 (μmol/L) (24W)	3.20 [2.51, 4.30]	3.20 [2.50, 4.20]	0.621
ALP (μmol/L) (24W)	82.50 [71.50, 99.50]	83.40 [69.25, 98.46]	0.904
GGT (U/L) (24W)	55.00 [35.50, 105.50]	48.00 [30.00, 87.07]	0.013
ALB (g/L) (24W)	44.00 [42.00, 45.30]	43.90 [42.00, 45.60]	0.94
GLO (g/L) (24W)	29.00 [26.83, 32.00]	29.00 [26.50, 32.00]	0.771
NEU(10^9^/L) (24W)	1.58 [1.21, 2.17]	1.64 [1.27, 2.23]	0.242
PLT (10^9^/L) (24W)	114.00 [91.00, 152.50]	117.00 [94.00, 152.00]	0.649
Hb(g/L) (24W)	135.00 [122.50, 146.50]	134.00 [121.00, 146.00]	0.671

Categorical data are presented as frequency (percentage). Normally distributed continuous variables are expressed as mean (standard deviation) and compared using the independent samples t-test. Non-normally distributed variables are summarized as median (interquartile range) and compared using the Mann-Whitney U test.

### 3.2 Variable selection

To identify robust predictors of response to interferon-based therapy, this study employed five distinct machine learning-based feature selection algorithms: Random Forest: The results identified the following ten top-ranking features: HBsAg (24W), HBsAg (0W), HBsAg_desc, ALT (24W), GGT (24W), DBIL (24W), HBcAb_desc, HBeAb (24W), HBeAg_desc, and HBcAb (0W), as detailed in Supplementary Table S2. LASSO Regression: The final model retained the following variables: HBsAg (24W), HBeAb (24W), ALT (24W), TBIL (24W), ALP (24W), Age, and Gender, as detailed in Supplementary Table S3. SVM-RFE: The results identified the following top features: HBsAg (24W), HBsAg (0W), HBsAg_desc, HBeAg_desc, HBcAb (0W), HBeAg(24W), GGT (0W), HBeAb (24W),HBeAb_desc, and HBeAg(0W), as presented in Supplementary Table S4. Elastic Net: The algorithm identified the following key variables: HBsAg (0W), HBsAg (24W), and HBsAg_desc, as detailed in Supplementary Table S5. XGBoost: The top 10 variables were: HBsAg (24W), HBsAg (0W), HBsAg_desc, ALT (24W), DBIL (24W), GGT (24W), HBeAb (24W), ALP (24W), HBcAb (0W), and TBIL (24W), as detailed in Supplementary Table S6.

To enhance model generalizability and clinical interpretability, we defined the optimal predictor set as variables selected by at least two out of the five feature selection algorithms, which yielded 11 key predictors (Supplementary Table S7). This consensus approach is visually summarized in a Venn diagram (Supplementary Figure S1). The final predictors were: ALP (24W), ALT (24W), DBIL (24W), GGT (0W), HBcAb (0W), HBeAb (24W), HBeAg_desc, HBsAg (0W), HBsAg_desc, HBsAg (24W), and TBIL (24W). Collectively, this set captures baseline status, on-treatment levels at mid-therapy, and early kinetic changes, thereby providing multi-dimensional predictive information.

### 3.3 Comparative performance of multiple machine learning models

**FIGURE 2:**
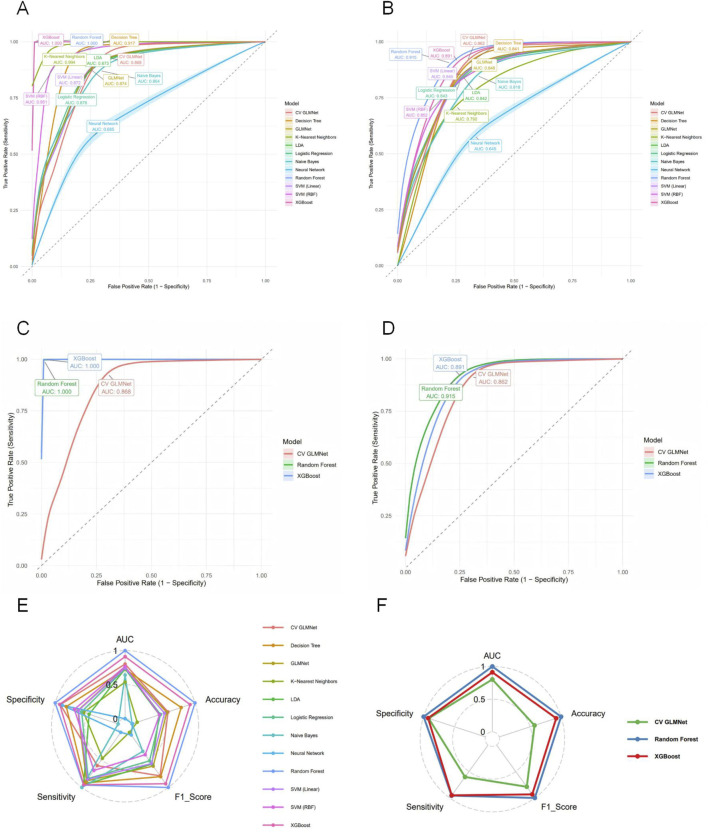
Evaluation of predictive performance for the twelve machine learning models. **(A)** ROC curves with 95% confidence intervals (shaded areas) for all models on the training set. **(B)** ROC curves for all models on the test set. **(C)** ROC curves for the top three performing models on the training set. **(D)** ROC curves for the top three models on the test set. **(E)** Radar plot comparing six performance metrics across all models on the test set. **(F)** Radar plot highlighting the balanced multi-metric performance of the top three models (ranked by test set AUC). The models are distinguished by color in all panels. The top three models, Random Forest, XGBoost, and CV GLMNet, consistently demonstrated superior and stable discriminative ability across both.

Based on the selected set of 11 key predictors, we constructed 12 distinct machine learning models, including Logistic Regression, LDA, Decision Tree, Random Forest, XGBoost, SVM (RBF), SVM (Linear), k-Nearest Neighbors, Neural Network, Naive Bayes, GLMNet, and CV GLMNet. The performance of all 12 models was rigorously evaluated on both the training and validation sets.The evaluation metrics included the Area Under the Curve (AUC), accuracy, sensitivity, specificity, precision, and F1-score. Results for each metric are reported as the mean values derived from 500 effective bootstrap samples (Supplementary Table S8). The Receiver Operating Characteristic (ROC) curve was used to assess the discriminative ability of the predictive models. These curves were plotted with 1-specificity on the x-axis and sensitivity on the y-axis, illustrating the performance for both the training and validation sets.In the training set, all models except Neural Network (AUC = 0.685 ± 0.168) demonstrated strong performance (AUC >0.85), as shown in Figure 2A. A comprehensive analysis of the ROC curves from the test set (Figure 2B) and the multi-metric radar chart (Figure 2E) identified three top-performing models: Random Forest (AUC = 0.915 ± 0.020), XGBoost (AUC = 0.891 ± 0.026), and CV GLMNet (AUC = 0.862 ± 0.029) (Figures 2C,D). In contrast, models such as k-Nearest Neighbors and Neural Network showed marked performance degradation on the validation set, with their ROC curves deviating substantially from the upper-left corner, indicating poorer generalizability.

### 3.4 Analysis of top-performing models

**FIGURE 3:**
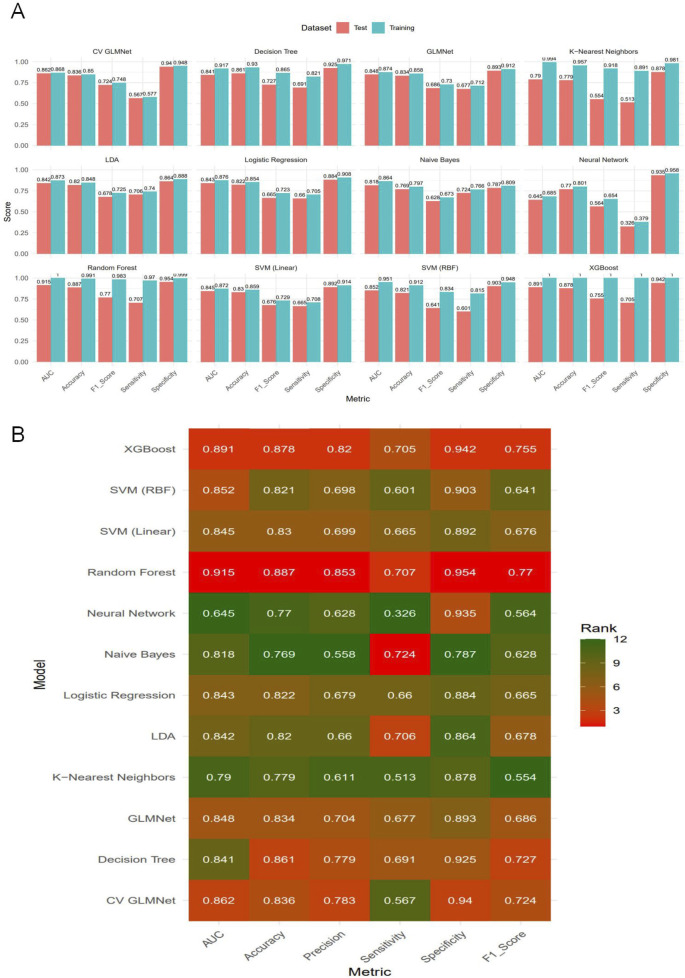
Comparative performance evaluation of the twelve machine learning models.**(A)** Bar plot displaying the bootstrap mean estimates of five performance metrics (AUC, Accuracy, F1-Score, Sensitivity, Specificity) for each model on both the training and test sets. **(B)** Heatmap summarizing the model rankings based on bootstrap mean performance on the test set. Color intensity corresponds to the metric score, with darker shades indicating higher values. The top-performing models—Random Forest, CV GLMNet, and XGBoost—consistently achieve superior and balanced results across most evaluation metrics in the test set.

**FIGURE 4:**
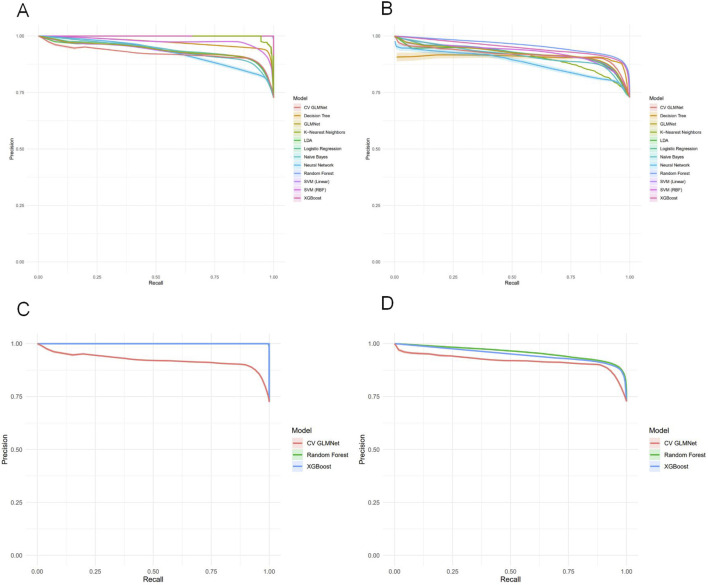
Precision-Recall analysis of machine learning models for HBsAg seroclearance prediction. **(A)** Precision-Recall curves with 95% confidence intervals for all twelve models on the training set. **(B)** Precision-Recall curves with 95% confidence intervals for all models on the test set. **(C)** Comparative Precision-Recall analysis of the top three performing models (Random Forest, XGBoost, and CV GLMNet) on the training set. **(D)** Comparative Precision-Recall analysis of the top three performing models (Random Forest, XGBoost, and CV GLMNet) on the test set.

Among the 12 machine learning models evaluated, Random Forest, XGBoost, and CV GLMNet consistently outperformed the others. The discriminative performance of Random Forest on the validation set was as follows: AUC = 0.915 ± 0.020, accuracy = 0.887 ± 0.019, precision = 0.853 ± 0.062, sensitivity = 0.707 ± 0.066, specificity = 0.954 ± 0.021, and F1-score = 0.770 ± 0.042. XGBoost achieved: AUC = 0.891 ± 0.026, accuracy = 0.878 ± 0.020, precision = 0.820 ± 0.064, sensitivity = 0.705 ± 0.063, specificity = 0.942 ± 0.023, and F1-score = 0.755 ± 0.043. CV GLMNet yielded: AUC = 0.862 ± 0.029, accuracy = 0.836 ± 0.063, precision = 0.783 ± 0.089, sensitivity = 0.567 ± 0.271, specificity = 0.940 ± 0.041, and F1-score = 0.724 ± 0.061 (Figures 3A,B).The Precision-Recall (PR) curves, plotted for both training and validation sets, demonstrated that CV GLMNet, Random Forest, and XGBoost all maintained high and stable predictive performance. Their PR curves were positioned close to the upper-right corner and exhibited narrow 95% confidence intervals, indicating a strong and reliable balance between precision and recall (Figures 4A–D).Notably, the Random Forest model achieved the highest AUC on the validation set (Figures 3A,B) and showed a balanced performance across accuracy, sensitivity, specificity, and F1-score (Figure 2F). Its performance on the validation set was comparable to that on the training set, with no significant overfitting observed. This demonstrates superior generalizability, establishing Random Forest as the optimal model for predicting HBsAg seroclearance.

### 3.5 Clinical utility assessment of the machine learning models

**FIGURE 5:**
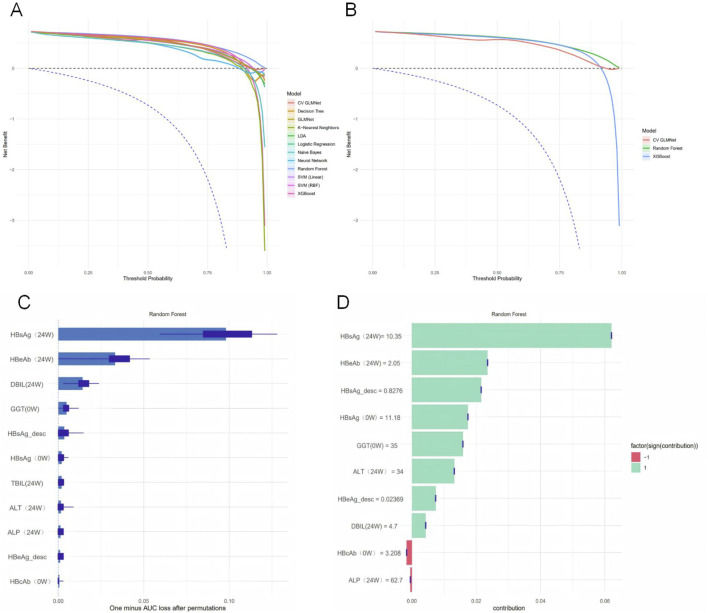
Model interpretability and clinical utility assessment. **(A)** Net benefit of all twelve models across a range of threshold probabilities, with 95% confidence intervals derived from bootstrap sampling. **(B)** Net benefit of the top three performing models (Random Forest, XGBoost, and CV GLMNet). The Random Forest model demonstrated superior performance. **(C)** Permutation Importance plot showing the mean decrease in model performance (1 - AUC) after randomly shuffling each feature. **(D)** SHAP summary plot displaying the magnitude and direction of feature contributions for the first 10 samples.

This study evaluated the clinical utility of the twelve machine learning models using Decision Curve Analysis (DCA), with 95% confidence intervals calculated to reflect the reproducibility of the results. As summarized in Table 5, the threshold corresponding to the maximum net benefit was 0.01 for all models. The Random Forest model demonstrated the highest Average Net Benefit Index (ANDI) value of 0.547 and the widest range of positive threshold probabilities (0.01–0.99). These results indicate its high clinical application value, supporting its use in guiding decisions on whether to continue PEG-IFNα-2b therapy in CHB patients.Decision curves were plotted to illustrate the relationship between decision thresholds (ranging from 0.00 to 1.00) and net benefit (ranging from −0.2–0.6) across the machine learning models. Over a wide range of threshold probabilities, the net benefit of the three top-performing models—Random Forest, XGBoost, and CV GLMNet—was consistently superior to that of the other models and remained higher than the “treat-all” or “treat-none” strategies, demonstrating robust clinical discriminative ability. Among them, the Random Forest model exhibited greater adaptability to threshold variations, as evidenced by its smoother curve and stable performance across different intervention scenarios. This suggests that Random Forest is particularly well-suited for balancing benefits and risks in clinical decision-making regarding antiviral therapy for hepatitis B (Figures 5A,B).

**TABLE 5:** Decision curve analysis (DCA) of the twelve machine learning models.

Model	ANDI	Max_Net_Benefit	Threshold_at_Max	Positive_Threshold_Range	N_Valid_Bootstraps
Logistic regression	0.4732	0.7262	0.01	0.01–0.93	500
LDA	0.4712	0.7264	0.01	0.01–0.93	500
Decision tree	0.491	0.7221	0.01	0.01–0.90	500
Random forest	0.547	0.7271	0.01	0.01–0.99	500
XGBoost	0.4528	0.7238	0.01	0.01–0.91	500
SVM (RBF)	0.4881	0.7271	0.01	0.01–0.94	500
SVM (linear)	0.4772	0.7268	0.01	0.01–0.94	500
K-nearest neighbors	0.3568	0.7166	0.01	0.01–0.89	500
Neural network	0.4116	0.7265	0.01	0.01–0.88	500
Naive bayes	0.3954	0.7173	0.01	0.01–0.90	500
GLMNet	0.4799	0.7267	0.01	0.01–0.93	500
CV GLMNet	0.4755	0.7271	0.01	0.01–0.93	500

To quantify the contribution of each predictor to the performance of the Random Forest model, we employed both the Permutation Importance method and SHAP analysis to assess variable importance (Figures 5C,D). The Permutation Importance results revealed significant differences in the importance of the 11 predictive features. HBsAg (24W) was identified as the most influential feature for model predictions, indicating that shuffling its values led to the greatest decrease in the model’s AUC. This establishes it as a core variable for maintaining the model’s discriminative ability. The following five features also provided substantial support for model performance: HBeAb (24W), DBIL (24W), GGT (0W), HBsAg_desc, and HBsAg (0W).Based on the SHAP analysis, using the shapviz package in R to compute Shapley values for features, a bar plot was generated to illustrate the contribution of the top 10 features to the model’s predictions in the Random Forest model. The ranking of feature contributions was as follows: HBsAg (24W) > HBeAb (24W) > HBsAg_desc > HBsAg (0W) > GGT (0W) > ALT (24W) > HBeAg_desc > DBIL (24W) > HBcAb (0W) > ALP (24W). Notably, HBcAb (0W) and ALP (24W) exhibited a negative predictive effect on HBsAg seroclearance, while the remaining variables showed positive predictive effects. Integrating the findings from both analytical methods, we conclude that HBsAg (24W), HBeAb (24W), GGT (0W), HBsAg_desc, and HBsAg (0W) are the key variables in the Random Forest model.

### 3.6 Predictive performance of individual variables

**FIGURE 6:**
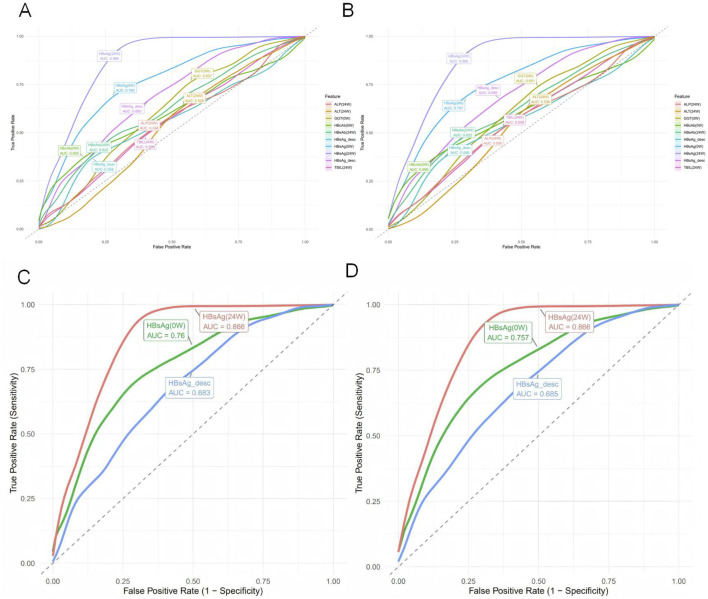
Univariate predictive performance of individual variables for HBsAg seroclearance. **(A)** Receiver operating characteristic (ROC) curves for the top 10 individual predictors on the training set, with area under the curve (AUC) values indicated. **(B)** ROC curves for the top 10 individual predictors on the test set. **(C)** ROC curves of the top three univariate predictors (HBsAg (24W), HBsAg (0W), and HBsAg_desc) on the training set. **(D)** ROC curves of the top three univariate predictors on the test set.

To compare the predictive performance between the machine learning models and individual variables, we conducted a univariate analysis of the 11 predictor variables on the test set, as detailed in Supplementary Table S9. ROC curves were plotted for the top ten individual features (Figures 6A,B). HBsAg (24W) was identified as the single most predictive feature, achieving an AUC of 0.866 ± 0.030, accuracy of 0.850 ± 0.025, precision of 0.723 ± 0.071, sensitivity of 0.736 ± 0.052, specificity of 0.893 ± 0.031, and an F1-score of 0.727 ± 0.046 on the validation set. Other valuable single features, in descending order of AUC, were HBsAg (0W) (AUC = 0.757 ± 0.032) and HBsAg_desc (AUC = 0.685 ± 0.036) (Figures 6C,D). In contrast, most liver function indicators—such as GGT (0W), TBIL (24W), ALP (24W), ALT (24W), and DBIL (24W)—demonstrated low predictive utility (AUC <0.6), indicating their limited value when used alone.

**FIGURE 7:**
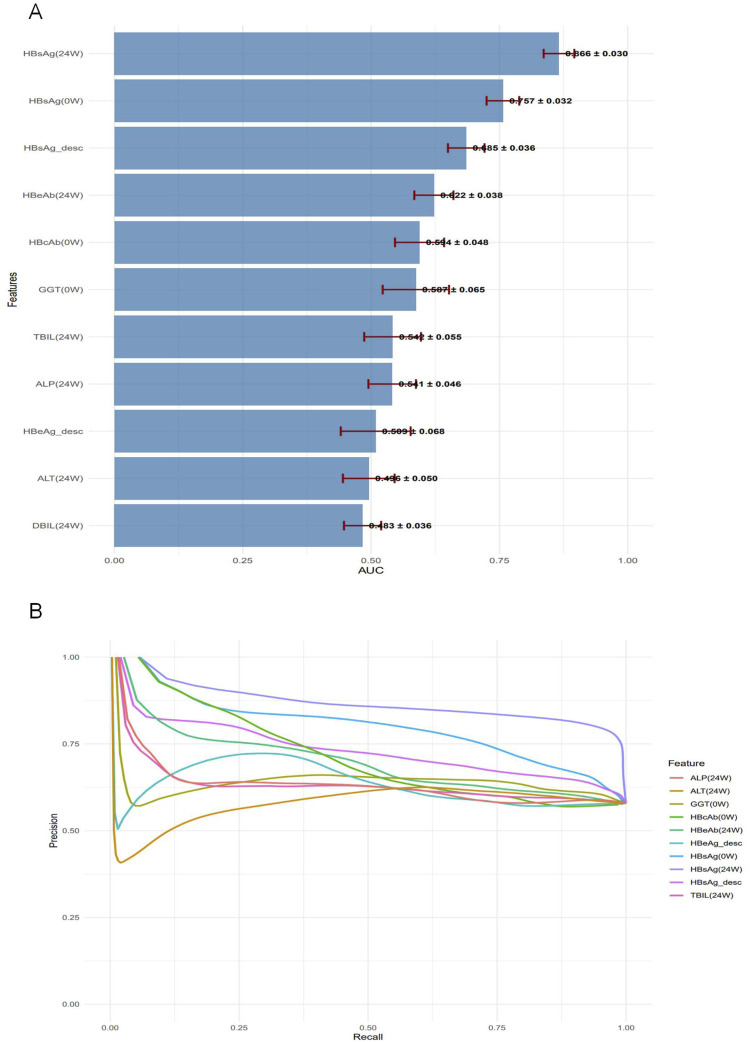
Performance assessment of individual predictors for HBsAg seroclearance. **(A)** Bar plot displaying the test set AUC values (mean ± standard deviation) of the univariate predictors, ranked in descending order. **(B)** Precision-Recall curves for the top 10 univariate predictors, illustrating their precision across the full range of recall values.

The univariate analysis in the test set was ranked by AUC in descending order (Figure 7A). Precision–Recall (PR) curves of the top 10 individual variables are plotted with Recall on the x-axis and Precision on the y-axis, illustrating the precision of each variable at different recall levels (Figure 7B). The PR curve for HBsAg (24W) was positioned closest to the upper-right corner, confirming its status as the best-performing single-variable predictor. However, this curve also exhibited noticeable fluctuations, reflecting its sensitivity to variations in a single feature. A sharp decline in precision was frequently observed at medium recall levels (0.4–0.6). In contrast, the Random Forest model demonstrated markedly superior and stable performance: its precision remained consistently high (mostly above 0.75) across the entire recall range (0–1) and was maintained at a high level even at high recall values (0.7–0.8), resulting in a much smoother and more robust curve. The Random Forest model, by integrating multiple predictive variables, accurately identifies patients with a high likelihood of achieving HBsAg seroclearance. This precise stratification helps prevent two critical clinical scenarios: firstly, avoiding the premature discontinuation of therapy in potential responders due to misclassification as “non-responders,” which could otherwise lead to a missed cure opportunity; and secondly, preventing the continuation of ineffective treatment in true non-responders misclassified as “responders” thereby reducing unnecessary PEG-IFNα-2b-related side effects and economic burdens (Table 6).

**TABLE 6:** Performance comparison between the random forest model and the univariate model (HBsAg at 24 Weeks).

Model_Type	Name	AUC	Accuracy	F1_Score
Multivariable	Random forest	0.915 ± 0.020	0.887 ± 0.019	0.770 ± 0.042
Univariable	HBsAg (24W)	0.866 ± 0.030	0.850 ± 0.025	0.727 ± 0.046

## 4 Discussion

This study integrated dynamic serological indicators from CHB patients with machine learning algorithms to develop and validate a predictive model for HBsAg seroclearance following PEG-IFNα-2b therapy. The main findings are as follows:

### 4.1 Clinical significance of key predictor variables

In this study, following data cleaning and the exclusion of patients with excessive missing data, the final dataset comprised 31 predictor variables spanning demographic and clinical characteristics, virological markers, liver function indices, and routine blood test parameters. These variables were subsequently processed using five distinct machine learning feature selection methods, which identified 11 optimal predictors. These 11 key variables were used to construct 12 machine learning models. Among them, the Random Forest model demonstrated the best performance (AUC = 0.915 ± 0.020) and surpassed the predictive capability of any single variable. Through Permutation Importance and SHAP analysis, the following variables were identified as the most influential within the Random Forest model: HBsAg (24W), HBeAb (24W), GGT (0W), HBsAg_desc, and HBsAg (0W).

HBsAg (24W) emerged as the most pivotal predictor in this study, achieving an AUC of 0.866 in the univariate analysis on the validation set and consistently ranking as the primary feature across multiple machine learning models. The quantitative HBsAg level serves as a key surrogate marker for the transcriptional activity of hepatitis B virus cccDNA. Both a lower baseline HBsAg level and a lower level at week 24 are strongly associated with a higher probability of HBsAg seroclearance at week 48 of interferon-based therapy (Tan et al., 2025; Wen et al., 2024). Furthermore, the magnitude of HBsAg decline or its absolute value at week 24 has been well-established as a robust predictor of response to interferon treatment (Jiang et al., 2024). Furthermore, incorporating the dynamic change in HBsAg (log_10_IU/mL) from baseline to week 24 further enhanced the model’s performance, underscoring that kinetic indicators provide a more reflective measure of treatment response trends than single time-point measurements (Reijnders et al., 2011).

During antiviral therapy, seroconversion of HBeAg at week 24—defined as the loss of HBeAg accompanied by the appearance of HBeAb—serves as a powerful predictor of favorable long-term outcomes. This serological change signifies the effective activation of the patient’s immune system, enabling it to better recognize and clear HBV-infected hepatocytes. A robust and effective cellular immune response is a prerequisite for achieving HBsAg seroclearance (Liaw et al., 2012).

This study identified a significant association between baseline GGT levels and HBsAg seroclearance. In prior research, low baseline GGT has been established as an independent predictor of sustained virological response in chronic hepatitis C treatment. A lower level of GGT indicates a significant reduction in liver inflammation and bile stasis, suggesting a relative preservation of liver cell function, which may better support the antiviral immune response induced by the combination of PEG-IFN and ribavirin (Akuta et al., 2007; Dogan et al., 2014). Furthermore, serum GGT has been proposed as a potential biomarker for predicting the activation of antiviral immunity and HBeAg seroconversion in HBeAg-positive chronic hepatitis B patients receiving nucleos(t)ide analogue (NAs) therapy (Huang, 2015). However, the role of GGT levels in predicting outcomes specifically for CHB patients treated with PEG-IFNα-2b requires further investigation and validation.

### 4.2 Superior performance of the machine learning model

In this study, the Random Forest (RF) model demonstrated superior performance. RF, an ensemble learning algorithm introduced by Breiman in 2001, operates on the core principle of “integrating votes from multiple decision trees” to model and predict complex data (Breiman, 2001). Its advantages lie in its capability to handle non-linear relationships and feature interactions, making it particularly suitable for modeling the complex associations between serological markers and treatment response (Jiang et al., 2009). Furthermore, its robustness to outliers and missing values reduces the impact of clinical data quality on model performance (Kokla et al., 2019). Permutation Importance, a model-agnostic tool for assessing feature significance, evaluates the importance of a variable by randomly shuffling its values in the validation dataset and observing the resultant decline in model performance metrics (e.g., accuracy, AUC). A greater decrease in performance indicates a higher importance of the feature to the model’s predictions. This method does not rely on the internal structure or parameters of any specific model, directly quantifying the feature’s contribution to the actual predictive performance (Fisher et al., 2019). SHAP values quantify the contribution of each feature to individual predictions, thereby assisting clinicians in understanding the model’s decision-making process and addressing the challenge of deploying traditional “black-box” models in clinical practice. By interpreting feature contributions through SHAP analysis, the model’s clinical interpretability is significantly enhanced (Lundberg and Lee, 2017; Shu et al., 2025).The combination of Permutation Importance and SHAP analysis provides complementary insights—from global feature importance to local explanations—consistently identifying HBsAg (24W) as the core predictor, with HBeAb (24W), HBsAg_desc, HBsAg (0W), and GGT (0W) as important supporting variables.For future clinical translation, a user-friendly predictive tool (such as a nomogram or a mobile application) could be developed to transform the Random Forest model into an accessible decision-support instrument for clinicians. Furthermore, prospective interventional studies are warranted to validate whether “RF model-guided therapy for CHB” can ultimately improve the rate of HBsAg seroclearance and reduce overall treatment costs.

### 4.3 Clinical application value of the machine learning model

The Random Forest model developed in this study can predict the likelihood of eventual HBsAg seroclearance in CHB patients as early as 24 weeks after initiating PEG-IFNα-2b therapy. Its clinical application includes several key aspects: For patients predicted as “non-achievers,” clinicians can promptly adjust the treatment strategy (e.g., switching to nucleos(t)ide analogues), thereby avoiding ineffective therapy and unnecessary drug-related adverse events. For those predicted as “achievers”, the positive prediction can reinforce treatment confidence and improve adherence. Furthermore, since all required variables (such as HBsAg and GGT) are routinely measured in standard clinical practice, the model can be implemented without additional testing, facilitating its widespread adoption.

### 4.4 Clinical translation and future directions

To facilitate the clinical translation of our predictive model, we have outlined a clear roadmap for subsequent research. First, we will precisely calibrate the decision thresholds for predicted probabilities using a prospective cohort to establish a risk stratification framework categorizing patients as “high-, intermediate-, and low-probability responders.” Based on this, a user-friendly clinical decision support tool will be developed to enable instant input of patient indicators and automatic output of stratification-based recommendations. Ultimately, a multicenter randomized controlled trial will be conducted to validate the effectiveness of this decision-making workflow, comparing the HBsAg seroclearance rates and medical costs between the “model-guided strategy” group and the “standard management” group. This systematic effort aims to transition our model from prediction to clinical practice.

## 5 Study limitations

However, this study has several limitations. First, the predictive model did not incorporate several potentially significant variables, including HBV genotype, HBV RNA, and host genetic polymorphisms (e.g., IL28B genotype). Previous research has established that patients with HBV genotype B exhibit a more pronounced decline in HBsAg and less rebound during interferon therapy, suggesting a more sustained immune response (Brunetto et al., 2009); In contrast, patients with HBV genotype D demonstrate a lower probability of achieving a sustained virological response, regardless of baseline ALT levels or HBV DNA load, identifying genotype D as an independent predictor of poor treatment outcome (Buster et al., 2009).Furthermore, small interfering RNA (siRNA) therapies targeting HBV RNA represent a promising strategy for achieving functional cure, with the substantial HBsAg reduction they induce being a key predictor of subsequent HBsAg seroclearance (Yuen et al., 2024). Additionally, IL28B gene polymorphisms have been significantly associated with HBsAg clearance (OR = 15.534, 95% CI: 1.998–120.777, P < 0.001) (Geng et al., 2024; Seto et al., 2013).Other functional biomarkers, including miR-548c-3p (Lin et al., 2023)and CXCL13 (Luo et al., 2023), have also been linked to HBsAg clearance. Future studies should incorporate these features to further refine the model’s accuracy; Second, the outcome measure was limited to “HBsAg seroclearance at 48 weeks of treatment” and was not linked to long-term clinical outcomes (e.g., the 5-year risk of hepatocellular carcinoma). Extending the follow-up period is necessary to comprehensively assess the prognostic implications.Furthermore, the model was only internally validated and lacked an independent external validation cohort. Both the training and validation data were sourced from the OASIS project, which may introduce data bias or overfitting. Consequently, the model’s generalizability to other independent cohorts (e.g., from different regions or using different testing platforms) and specific subgroups (such as patients with HBV genotype D) remains unverified, potentially affecting the reliability of its clinical application.

## 6 Conclusion

The Random Forest model, constructed based on serological data from CHB patients, effectively predicts the HBsAg seroclearance rate at 48 weeks of PEG-IFNα-2b therapy. Key predictive variables identified include HBsAg (24W), HBeAb (24W), GGT (0W), HBsAg_desc, and HBsAg (0W). The model demonstrates strong discriminative ability and clinical utility, providing valuable data-driven support for clinicians to develop individualized interferon treatment strategies.

## Data Availability

The raw data supporting the conclusions of this article will be made available by the authors, without undue reservation.
