# Supplementary figures and images for: A machine learning-based predictive model for 48-week hepatitis B surface antigen seroclearance in chronic hepatitis B patients treated with pegylated interferon α-2b: prediction at week 24
Source: Front Cell Dev Biol. 2025 Nov 26;13:1734654. doi: 10.3389/fcell.2025.1734654 (PMC12689909; doi:10.3389/fcell.2025.1734654)

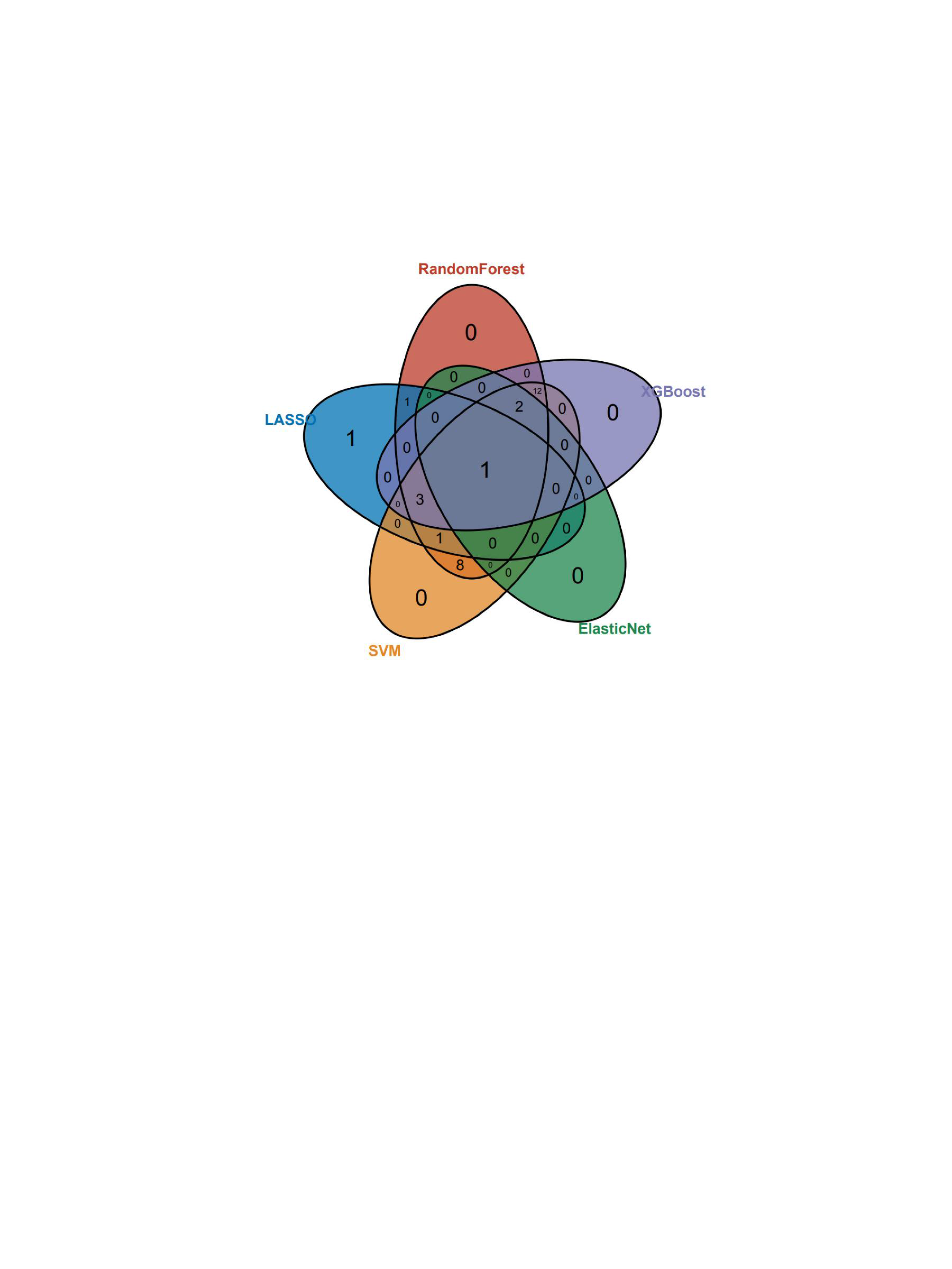

Supplement: Supplementary file 1 [file Image1.tif]
