# Supplementary material for: A machine learning-based predictive model for 48-week hepatitis B surface antigen seroclearance in chronic hepatitis B patients treated with pegylated interferon α-2b: prediction at week 24
Source: Front Cell Dev Biol. 2025 Nov 26;13:1734654. doi: 10.3389/fcell.2025.1734654 (PMC12689909; doi:10.3389/fcell.2025.1734654)
Supplement: Supplementary file 2 [file Table1.doc]

Supplementary Table 1. Categorization and description of predictor variables included in the analysis.

| **Variable Category** | **Variable Name** | **Description** |
| --- | --- | --- |
| 1. Demographic and clinical characteristics | Age | Age (years) |
|  | Gender | Sex (Male=1/Female=0) |
|  | BMI | Body Mass Index |
|  | LC | Liver Cirrhosis (Yes=1/No=0) |
|  | Family history | Family History of HBV (Yes=1/No=0) |
| 2. Virological Markers | HBsAg(0W) | Baseline HBsAg level |
|  | HBsAg(24W) | HBsAg level at Week 24 |
|  | HBsAg_desc | Change in HBsAg from baseline to Week 24 |
|  | HBeAg(0W) | Baseline HBeAg level |
|  | HBeAg(24W) | HBeAg level at Week 24 |
|  | HBeAg_desc | Change in HBeAg from baseline to Week 24 |
|  | HBeAb(0W) | Baseline HBeAb level |
|  | HBeAb(24W) | HBeAb level at Week 24 |
|  | HBeAb_desc | Change in HBeAb from baseline to Week 24 |
|  | HBcAb(0W) | Baseline HBcAb level |
|  | HBcAb(24W) | HBcAb level at Week 24 |
|  | HBcAb_desc | Change in HBcAb from baseline to Week 24 |
| 3. Liver Function Indices | ALT(0W) | Baseline ALT |
|  | ALT(24W) | ALT at Week 24 |
|  | AST(0W) | Baseline AST |
|  | AST(24W) | AST at Week 24 |
|  | GGT(0W) | Baseline GGT |
|  | GGT(24W) | GGT at Week 24 |
|  | TBIL(0W) | Baseline Total Bilirubin |
|  | TBIL(24W) | Total Bilirubin at Week 24 |
|  | DBIL(0W) | Baseline Direct Bilirubin |
|  | DBIL(24W) | Direct Bilirubin at Week 24 |
|  | ALP(0W) | Baseline Alkaline Phosphatase |
|  | ALP(24W) | Alkaline Phosphatase at Week 24 |
| 4. Routine Blood Test Indices | PLT(0W) | Baseline Platelet Count |
|  | PLT(24W) | Platelet Count at Week 24 |

**Supplementary Table 2. Features Selected by the Random Forest Algorithm.**

| **Feature** | **MeanDecreaseGini** |
| --- | --- |
| HBsAg(24W) | 52.50125335 |
| HBsAg(0W) | 16.69257096 |
| HBsAg_desc | 13.40446719 |
| ALT(24W) | 5.54491047 |
| GGT(24W) | 5.412224717 |
| DBIL(24W) | 5.376270202 |
| HBcAb_desc | 4.78590526 |
| HBeAb(24W) | 4.781331739 |
| HBeAg_desc | 4.448242927 |
| HBcAb(0W) | 4.206646057 |

Supplementary Table 3. **Features Selected by LASSO Regression.**

| **obs** | **coef** |
| --- | --- |
| HBsAg(24W) | 0.308332327 |
| HBeAb(24W) | 0.021008196 |
| ALT(24W) | -0.000805194 |
| TBIL(24W) | 0.005493465 |
| ALP(24W) | 0.002213635 |
| Age | 0.013881531 |
| Gender | -0.003478428 |

Supplementary Table 4. **Features Selected by the SVM-RFE Algorithm.**

| **Feature** | **Importance** |
| --- | --- |
| HBsAg(24W) | 0.48394837 |
| HBsAg(0W) | 0.184570685 |
| HBsAg_desc | 0.122566072 |
| HBeAg_desc | 0.060036501 |
| HBcAb(0W) | 0.053292176 |
| HBeAg(24W) | 0.051574329 |
| GGT(0W) | 0.047908381 |
| HBeAb(24W) | 0.042202849 |
| HBeAb_desc | 0.03632508 |
| HBeAg(0W) | 0.034167515 |

Supplementary Table 5. **Features** Selected by the Elastic Net Algorithm.

| **obs** | **coef** |
| --- | --- |
| HBsAg(0W) | 0.054715744 |
| HBsAg(24W) | 0.161581031 |
| HBsAg_desc | -0.043043271 |

Supplementary Table 6. Features Selected by the XGBoost Algorithm.

| **Feature** | **Gain** | **Cover** | **Frequency** |
| --- | --- | --- | --- |
| HBsAg(24W) | 0.457158508 | 0.238975614 | 0.159851301 |
| HBsAg(0W) | 0.064030571 | 0.071291037 | 0.042750929 |
| HBsAg_desc | 0.051245574 | 0.050293956 | 0.042750929 |
| ALT(24W) | 0.045446681 | 0.066878643 | 0.083643123 |
| DBIL(24W) | 0.038919576 | 0.080912451 | 0.057620818 |
| GGT(24W) | 0.034413264 | 0.039275557 | 0.053903346 |
| HBeAb(24W) | 0.034328342 | 0.064813091 | 0.035315985 |
| ALP(24W) | 0.027723507 | 0.049058564 | 0.042750929 |
| HBcAb(0W) | 0.026335015 | 0.046555369 | 0.044609665 |
| TBIL(24W) | 0.02327218 | 0.035398678 | 0.035315985 |

Supplementary Table 7. Complete list of features selected by each of the five machine learning algorithms.

| **RandomForest** | **LASSO** | **SVM** | **ElasticNet** | **XGBoost** |
| --- | --- | --- | --- | --- |
| HBsAg(24W) | HBsAg(24W) | HBsAg(24W) | HBsAg(0W) | HBsAg(24W) |
| HBsAg(0W) | HBeAb(24W) | HBsAg(0W) | HBsAg(24W) | HBsAg(0W) |
| HBsAg_desc | ALT(0W) | HBsAg_desc | HBsAg_desc | HBsAg_desc |
| ALT(24W) | TBIL(24W) | HBeAg_desc |  | ALT(24W) |
| GGT(0W) | ALP(24W) | HBcAb(0W) |  | DBIL(24W) |
| DBIL(24W) | Age | HBeAg(24W) |  | GGT(24W) |
| HBcAb_desc | Gender | GGT(0W) |  | HBeAb(24W) |
| HBeAb(24W) |  | HBeAb(24W) |  | ALP(24W) |
| HBeAg_desc |  | HBeAb_desc |  | HBcAb(0W) |
| HBcAb(0W) |  | HBeAg(0W) |  | TBIL(24W) |
| ALT(0W) |  | HBeAb(0W) |  | PLT(0W) |
| ALP(24W) |  | DBIL(24W) |  | HBcAb(24W) |
| GGT(24W) |  | HBcAb(24W) |  | HBeAb(0W) |
| AST(24W) |  | ALT(24W) |  | HBeAg_desc |
| AST(0W) |  | HBcAb_desc |  | HBeAg(24W) |
| HBeAb(0W) |  | ALT(0W) |  | PLT(24W) |
| HBeAg(24W) |  | AST(0W) |  | GGT(0W) |
| TBIL(24W) |  | ALP(24W) |  | AST(24W) |
| HBeAb_desc |  | DBIL(0W) |  |  |
| BMI |  | BMI |  |  |
| PLT(0W) |  | AST(24W) |  |  |
| Age |  | GGT(24W) |  |  |
| DBIL(0W) |  | PLT(0W) |  |  |
| HBcAb(24W) |  | ALP(0W) |  |  |
| TBIL(0W) |  | PLT(24W) |  |  |
| ALP(0W) |  | TBIL(24W) |  |  |
| HBeAg(0W) |  | TBIL(0W) |  |  |
| PLT(24W) |  |  |  |  |

Supplementary Table 8. **Performance Comparison of the Twelve Machine Learning Models on the Training and Test Sets.**

| **Dataset** | **Model** | **AUC** | **Accuracy** | **Sensitivity** | **Specificity** | **Precision** | **F1_Score** | **N_Valid_Bootstraps** |
| --- | --- | --- | --- | --- | --- | --- | --- | --- |
| Training | Logistic Regression | 0.876 ± 0.020 | 0.854 ± 0.020 | 0.705 ± 0.059 | 0.908 ± 0.016 | 0.743 ± 0.030 | 0.723 ± 0.041 | 500 |
| Training | LDA | 0.873 ± 0.020 | 0.848 ± 0.017 | 0.740 ± 0.053 | 0.888 ± 0.019 | 0.713 ± 0.025 | 0.725 ± 0.032 | 500 |
| Training | Decision Tree | 0.917 ± 0.023 | 0.930 ± 0.011 | 0.821 ± 0.044 | 0.971 ± 0.012 | 0.916 ± 0.031 | 0.865 ± 0.023 | 500 |
| Training | Random Forest | 1.000 ± 0.000 | 0.991 ± 0.004 | 0.970 ± 0.014 | 0.999 ± 0.002 | 0.997 ± 0.004 | 0.983 ± 0.008 | 500 |
| Training | XGBoost | 1.000 ± 0.000 | 1.000 ± 0.000 | 1.000 ± 0.000 | 1.000 ± 0.000 | 1.000 ± 0.000 | 1.000 ± 0.000 | 500 |
| Training | SVM (RBF) | 0.951 ± 0.011 | 0.912 ± 0.014 | 0.815 ± 0.040 | 0.948 ± 0.011 | 0.856 ± 0.028 | 0.834 ± 0.029 | 500 |
| Training | SVM (Linear) | 0.872 ± 0.020 | 0.859 ± 0.019 | 0.708 ± 0.071 | 0.914 ± 0.019 | 0.757 ± 0.030 | 0.729 ± 0.045 | 500 |
| Training | K-Nearest Neighbors | 0.994 ± 0.002 | 0.957 ± 0.008 | 0.891 ± 0.029 | 0.981 ± 0.007 | 0.947 ± 0.018 | 0.918 ± 0.017 | 500 |
| Training | Neural Network | 0.685 ± 0.168 | 0.801 ± 0.078 | 0.379 ± 0.373 | 0.958 ± 0.058 | 0.788 ± 0.138 | 0.654 ± 0.244 | 500 |
| Training | Naive Bayes | 0.864 ± 0.020 | 0.797 ± 0.027 | 0.766 ± 0.041 | 0.809 ± 0.031 | 0.601 ± 0.044 | 0.673 ± 0.038 | 500 |
| Training | GLMNet | 0.874 ± 0.020 | 0.858 ± 0.018 | 0.712 ± 0.060 | 0.912 ± 0.017 | 0.752 ± 0.028 | 0.730 ± 0.039 | 500 |
| Training | CV GLMNet | 0.868 ± 0.021 | 0.850 ± 0.053 | 0.577 ± 0.274 | 0.948 ± 0.032 | 0.815 ± 0.050 | 0.748 ± 0.051 | 500 |
| Test | Logistic Regression | 0.843 ± 0.029 | 0.822 ± 0.025 | 0.660 ± 0.071 | 0.884 ± 0.032 | 0.679 ± 0.071 | 0.665 ± 0.050 | 500 |
| Test | LDA | 0.842 ± 0.029 | 0.820 ± 0.025 | 0.706 ± 0.067 | 0.864 ± 0.035 | 0.660 ± 0.069 | 0.678 ± 0.046 | 500 |
| Test | Decision Tree | 0.841 ± 0.038 | 0.861 ± 0.027 | 0.691 ± 0.073 | 0.925 ± 0.036 | 0.779 ± 0.086 | 0.727 ± 0.052 | 500 |
| Test | Random Forest | 0.915 ± 0.020 | 0.887 ± 0.019 | 0.707 ± 0.066 | 0.954 ± 0.021 | 0.853 ± 0.062 | 0.770 ± 0.042 | 500 |
| Test | XGBoost | 0.891 ± 0.026 | 0.878 ± 0.020 | 0.705 ± 0.063 | 0.942 ± 0.023 | 0.820 ± 0.064 | 0.755 ± 0.043 | 500 |
| Test | SVM (RBF) | 0.852 ± 0.027 | 0.821 ± 0.024 | 0.601 ± 0.079 | 0.903 ± 0.029 | 0.698 ± 0.068 | 0.641 ± 0.052 | 500 |
| Test | SVM (Linear) | 0.845 ± 0.028 | 0.830 ± 0.024 | 0.665 ± 0.081 | 0.892 ± 0.033 | 0.699 ± 0.071 | 0.676 ± 0.050 | 500 |
| Test | K-Nearest Neighbors | 0.790 ± 0.031 | 0.779 ± 0.025 | 0.513 ± 0.070 | 0.878 ± 0.030 | 0.611 ± 0.072 | 0.554 ± 0.054 | 500 |
| Test | Neural Network | 0.645 ± 0.142 | 0.770 ± 0.061 | 0.326 ± 0.330 | 0.935 ± 0.080 | 0.628 ± 0.188 | 0.564 ± 0.222 | 500 |
| Test | Naive Bayes | 0.818 ± 0.038 | 0.769 ± 0.035 | 0.724 ± 0.066 | 0.787 ± 0.041 | 0.558 ± 0.066 | 0.628 ± 0.056 | 500 |
| Test | GLMNet | 0.848 ± 0.028 | 0.834 ± 0.024 | 0.677 ± 0.071 | 0.893 ± 0.031 | 0.704 ± 0.072 | 0.686 ± 0.049 | 500 |
| Test | CV GLMNet | 0.862 ± 0.029 | 0.836 ± 0.063 | 0.567 ± 0.271 | 0.940 ± 0.041 | 0.783 ± 0.089 | 0.724 ± 0.061 | 500 |

Supplementary Table 9. Predictive Performance of Individual Variables.

| **Dataset** | **Feature** | **AUC** | **Accuracy** | **Sensitivity** | **Specificity** | **Precision** | **F1_Score** | **N_Valid_Bootstraps** |
| --- | --- | --- | --- | --- | --- | --- | --- | --- |
| Test | HBsAg(24W) | 0.866 ± 0.030 | 0.850 ± 0.025 | 0.736 ± 0.052 | 0.893 ± 0.031 | 0.723 ± 0.071 | 0.727 ± 0.046 | 500 |
| Test | HBsAg(0W) | 0.757 ± 0.032 | 0.765 ± 0.026 | 0.347 ± 0.061 | 0.922 ± 0.030 | 0.634 ± 0.091 | 0.443 ± 0.055 | 500 |
| Test | HBsAg_desc | 0.685 ± 0.036 | 0.751 ± 0.025 | 0.216 ± 0.069 | 0.952 ± 0.031 | 0.662 ± 0.147 | 0.314 ± 0.075 | 500 |
| Test | HBeAb(24W) | 0.622 ± 0.038 | 0.727 ± 0.027 | 0.000 ± 0.000 | 1.000 ± 0.000 | NaN ± NA | NaN ± NA | 500 |
| Test | HBcAb(0W) | 0.594 ± 0.048 | 0.729 ± 0.026 | 0.001 ± 0.004 | 0.998 ± 0.008 | 0.077 ± 0.171 | 0.011 ± 0.019 | 500 |
| Test | GGT(0W) | 0.587 ± 0.065 | 0.729 ± 0.026 | 0.000 ± 0.000 | 1.000 ± 0.001 | 0.000 ± NA | 0.000 ± NA | 500 |
| Test | TBIL(24W) | 0.542 ± 0.055 | 0.729 ± 0.026 | 0.000 ± 0.000 | 1.000 ± 0.000 | NaN ± NA | NaN ± NA | 500 |
| Test | ALP(24W) | 0.541 ± 0.046 | 0.728 ± 0.028 | 0.000 ± 0.002 | 0.999 ± 0.002 | 0.032 ± 0.132 | 0.004 ± 0.015 | 500 |
| Test | HBeAg_desc | 0.509 ± 0.068 | 0.728 ± 0.028 | 0.000 ± 0.003 | 1.000 ± 0.002 | 0.472 ± 0.499 | 0.020 ± 0.021 | 500 |
| Test | ALT(24W) | 0.496 ± 0.050 | 0.726 ± 0.027 | 0.002 ± 0.008 | 0.995 ± 0.009 | 0.044 ± 0.109 | 0.009 ± 0.022 | 500 |
| Test | DBIL(24W) | 0.483 ± 0.036 | 0.728 ± 0.026 | 0.001 ± 0.004 | 1.000 ± 0.003 | 0.370 ± 0.381 | 0.029 ± 0.027 | 500 |
| Training | HBsAg(24W) | 0.866 ± 0.022 | 0.853 ± 0.016 | 0.740 ± 0.042 | 0.894 ± 0.016 | 0.721 ± 0.023 | 0.730 ± 0.027 | 500 |
| Training | HBsAg(0W) | 0.760 ± 0.025 | 0.770 ± 0.020 | 0.355 ± 0.063 | 0.923 ± 0.016 | 0.632 ± 0.044 | 0.452 ± 0.061 | 500 |
| Training | HBsAg_desc | 0.683 ± 0.027 | 0.758 ± 0.020 | 0.222 ± 0.080 | 0.957 ± 0.022 | 0.674 ± 0.087 | 0.325 ± 0.093 | 500 |
| Training | HBeAb(24W) | 0.623 ± 0.027 | 0.729 ± 0.021 | 0.000 ± 0.000 | 1.000 ± 0.000 | NaN ± NA | NaN ± NA | 500 |
| Training | GGT(0W) | 0.602 ± 0.045 | 0.728 ± 0.020 | 0.000 ± 0.000 | 1.000 ± 0.000 | NaN ± NA | NaN ± NA | 500 |
| Training | HBcAb(0W) | 0.598 ± 0.032 | 0.726 ± 0.021 | 0.001 ± 0.005 | 0.999 ± 0.004 | 0.201 ± 0.237 | 0.017 ± 0.020 | 500 |
| Training | TBIL(24W) | 0.554 ± 0.029 | 0.727 ± 0.019 | 0.000 ± 0.000 | 1.000 ± 0.000 | 0.333 ± NA | 0.014 ± NA | 500 |
| Training | ALP(24W) | 0.548 ± 0.027 | 0.728 ± 0.020 | 0.000 ± 0.003 | 1.000 ± 0.001 | 0.064 ± 0.216 | 0.007 ± 0.022 | 500 |
| Training | HBeAg_desc | 0.534 ± 0.055 | 0.730 ± 0.020 | 0.001 ± 0.007 | 1.000 ± 0.001 | 0.879 ± 0.231 | 0.036 ± 0.036 | 500 |
| Training | ALT(24W) | 0.523 ± 0.030 | 0.727 ± 0.019 | 0.004 ± 0.015 | 0.998 ± 0.004 | 0.256 ± 0.314 | 0.029 ± 0.044 | 500 |
| Training | DBIL(24W) | 0.520 ± 0.022 | 0.729 ± 0.021 | 0.001 ± 0.006 | 1.000 ± 0.001 | 0.856 ± 0.259 | 0.040 ± 0.025 | 500 |

Performance metrics are presented as mean ± standard deviation. NaN (Not a Number) and NA (Not Available) values for Precision and F1-score occur when no positive cases are predicted by the model in certain bootstrap samples. This typically happens when a single variable has extremely low sensitivity, resulting in no true positive or false positive predictions, which makes Precision and F1-score undefined.
